# A Rare Presentation of Knee Pain in a Patient With History of Lung Cancer

**DOI:** 10.7759/cureus.96329

**Published:** 2025-11-07

**Authors:** Juan Gabriel Jimenez Garcia, Juan Ramon Santos Rivera, Shruti Trehan, Achal Vaidya

**Affiliations:** 1 Internal Medicine, Florida International University, Miami, USA; 2 Internal Medicine, Ponce Health Sciences University, Ponce, PRI; 3 Hematology and Oncology, Miami Veterans Affairs Medical Center, Miami, USA; 4 Rheumatology, Miami Veterans Affairs Medical Center, Miami, USA

**Keywords:** adenocarcinoma lung, bone metastasis, hematology-oncology, internal medicine and rheumatology, rare metastases, rheumatoid arthritis

## Abstract

Intra-articular malignancies affecting the knee and hip joint are extraordinarily rare presentations of metastatic disease. We present the case of a 64-year-old woman with a history of psoriasis, psoriatic arthritis, and right lung cancer, who presented with worsening right knee pain initially attributed to a psoriatic arthritis flare secondary to immune checkpoint inhibitor therapy used as adjuvant treatment for her lung cancer. However, persistent and worsening symptoms despite discontinuation of the offending agent and use of corticosteroids and analgesics prompted further evaluation. Magnetic resonance imaging revealed a soft tissue mass in the intercondylar space, and subsequent positron emission tomography-computed tomography demonstrated hypermetabolic activity in the right knee and right ischial tuberosity. These findings led to a biopsy, which confirmed the diagnosis of intra-articular metastasis from lung adenocarcinoma. Our case highlights the importance of considering protean manifestations of metastatic malignancy in our differential diagnoses and always listening to our patient.

## Introduction

Lung cancer is currently the second most common cancer in the US and is associated with the highest number of cancer deaths in the US [1]. Risk factors include tobacco smoking, a family history of lung cancer, and occupational exposure to substances like silica, asbestos, heavy metals, and other hazardous agents. Adenocarcinoma is the most common histological subtype of non-small cell lung cancer, as well as the most frequent histology in non-smokers [2]. Despite multiple improvements in management, including molecular markers, such as epidermal growth factor receptor (EGFR) and anaplastic lymphoma kinase (ALK) targeted treatments as well as immune checkpoint inhibitors, that have resulted in improvement in lung cancer mortality, there remains a significant risk of metastases [3]. Common sites of lung cancer metastases include lymph nodes, bones, liver, brain, and intrapulmonary metastases [4]. Intra-articular metastases are exceedingly rare [5]. We present a rare case of knee monoarthropathy caused by metastatic lung adenocarcinoma, highlighting the diagnostic approach.

## Case presentation

A 64-year-old Hispanic woman, a former smoker with a history of psoriasis and untreated psoriatic arthritis, underwent longitudinal evaluation in the outpatient clinics from December 2023 to January 2024 for persistent right knee pain. She had been recently diagnosed with T1cN2 lung adenocarcinoma and had undergone right upper lobectomy, adjuvant chemotherapy, radiation, and initiation of immune checkpoint inhibitor therapy with PD-L1 inhibitor atezolizumab. Shortly after starting immunotherapy, she developed severe arthritis in multiple joints and a desquamating rash, which rheumatology and oncology attributed to a psoriatic arthritis flare triggered by atezolizumab. Her treatment included discontinuation of atezolizumab and initiation of corticosteroids and analgesics, leading to resolution of symptoms except for the persistent right knee pain.

**Figure 1:**
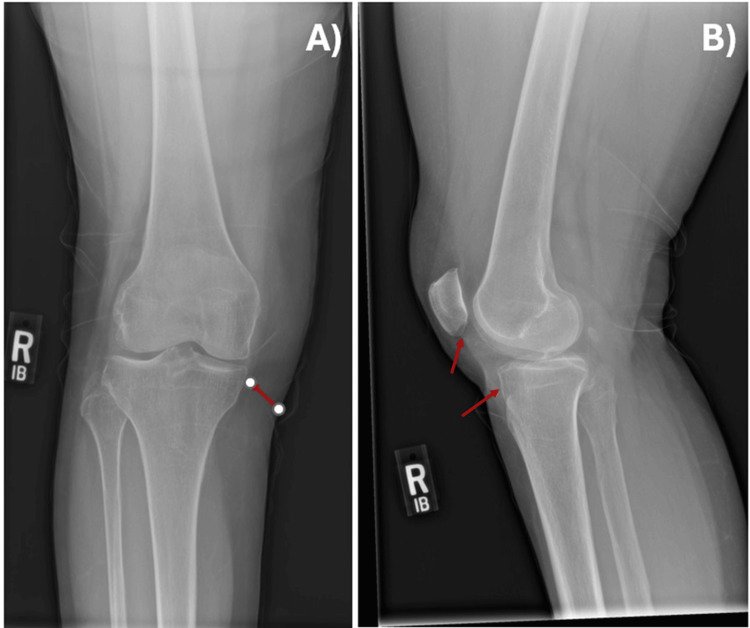
X-ray of the right knee in frontal (A) and lateral (B) views, with red arrows highlighting areas of cortical thinning.

The patient continued to experience unilateral knee pain, with the only notable lab abnormality being elevated alkaline phosphatase, initially attributed to cholestasis (Table 1). Due to persistent right knee pain, she was referred to physical therapy. A plain radiograph of the right knee was unremarkable except for osteopenia (Figure 1). As her knee pain worsened, causing a limp and affecting her mobility, she was referred to physical medicine and rehabilitation (PM&R) for steroid injections. To assist with her gait abnormality and improve safety, she was provided with a cane and a shower chair.

**Table 1:** Serum labwork done initially.

Test	Result	Normal range
White blood cell (WBC)	7.3 x 10^9^/L	4.5–11.0 x 10^9^/L
Hemoglobin (Hgb)	11.0 g/dL	12.1–15.1 g/dL
Hematocrit (Hct)	33.4%	36.1–44.3%
Platelet (Plt)	219 x 10^9^/L	150–450 x 10^9^/L
Alanine aminotransferase (ALT)	20 IU/L	7–56 IU/L
Aspartate aminotransferase (AST)	30 IU/L	10–40 IU/L
Alkaline phosphatase	220 IU/L	44–147 IU/L
Erythrocyte sedimentation rate (ESR)	64 mm/h	0–20 mm/h
C-reactive protein (CRP)	10.2 mg/L	0–3 mg/L

On physical examination, the patient exhibited a slight antalgic gait. There was mild, non-tender bony prominence of the metacarpophalangeal joints, with no swelling or tenderness in the proximal interphalangeal or distal interphalangeal joints, elbows, wrists, or metatarsophalangeal joints. No facial erythema or plaques were observed on the extremities. However, the right knee showed mild warmth, a small effusion, and reduced flexion. Additionally, the patient reported mild discomfort in the right hip with flexion.

**Figure 2:**
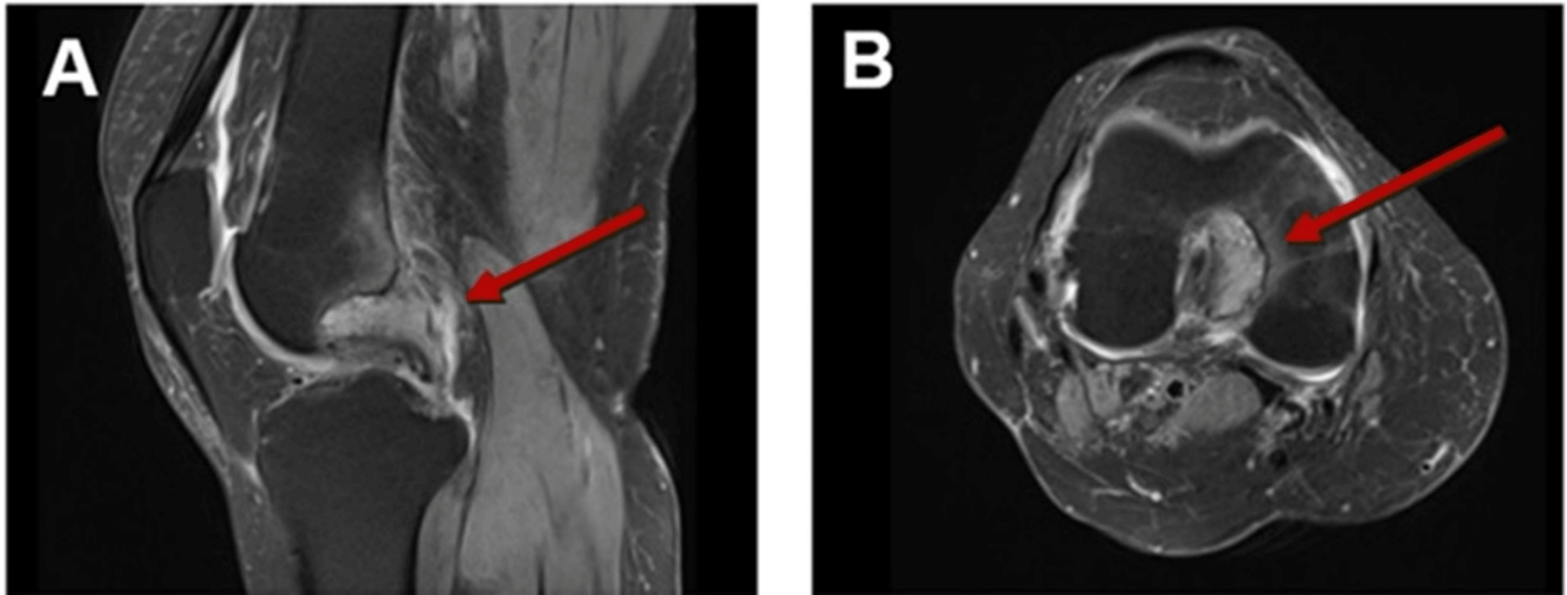
Magnetic resonance imaging without contrast of the right knee showing soft oval tissue mass in the medial intercondylar notch with adjacent bone erosion (red arrow).

**Figure 3:**
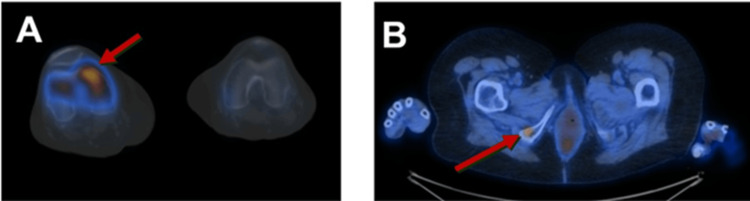
PET/CT scan showing hypermetabolic activity in the right knee (A) as well as a 7 mm hypermetabolic lesion in the right ischial tuberosity (B).

Due to the patient's persistent pain and refractoriness to all treatment modalities, a magnetic resonance imaging (MRI) with and without contrast of the right knee was ordered. The MRI revealed a soft tissue mass in the intercondylar space, causing cortical erosion and edema (Figure 2). The differential diagnosis based on imaging included nodular synovitis and pigmented villonodular synovitis. Given the suspicion of malignancy and to assess the status of her lung cancer, a PET/CT scan was also performed. The PET/CT demonstrated hypermetabolic activity in the right knee and the right ischial tuberosity (Figure 3). The PET/CT findings of abnormal uptake in the right intercondylar space and the right ischial tuberosity raised significant concerns for metastasis.

**Figure 4:**
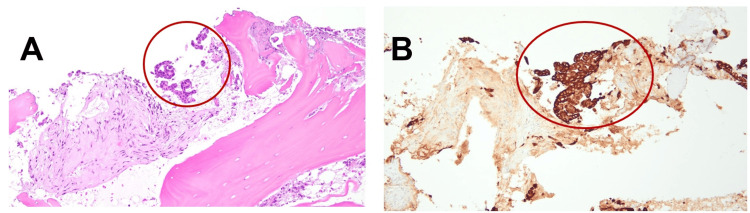
(A) H&E staining of epithelial cells infiltrating (within red circle); (B) IHC with anti-pancytokeratin antibody labeling respiratory epithelial cells (within red circle). IHC: immunohistochemistry.

Orthopedics was consulted for a biopsy, but due to the abnormal uptake in both the right intercondylar space and the right ischial tuberosity, the latter was deemed a more suitable biopsy site. A multidisciplinary meeting involving oncology, radiation oncology, and interventional radiology determined that the right ischial tuberosity was the preferred site for biopsy. Interventional radiology performed a bone biopsy at this location, and histopathology confirmed metastasis of lung adenocarcinoma (Figure 4). The timeline from her psoriatic arthritis flare induced by immunotherapy to the diagnosis of lung adenocarcinoma metastasis as the cause of her progressively debilitating knee pain was 12 months. The patient was restaged and started on a course of palliative radiation to the right knee to be followed by systemic therapy.

## Discussion

Lung cancer is one of the malignancies commonly associated with bone metastases and is often linked to a poor prognosis [4]. Around 20-30% of patients with non-small cell lung cancer (NSCLC) present with bone metastases, typically affecting the axial skeleton [6]. Symptoms of bone metastases are common, with most patients experiencing significant pain during the disease course. Lung adenocarcinoma carries a higher risk of bone metastases compared to other pathological types, with spinal metastases occurring in approximately 79% of patients [7]. Across all lung cancer subtypes, the most common site of bone metastasis is the spine (>50%), followed by the ribs (50%), pelvis (20%), femur (15%), and sternum (15%), highlighting a predilection for the axial skeleton [8].

Metastasis involving synovium is a highly unusual phenomenon, with lung adenocarcinoma being the most commonly encountered type of synovial metastasis [9]. Approximately 48 cases of synovial metastasis from distinct cancers have been documented in the literature, highlighting the rarity of this condition [9]. The most common symptoms of knee involvement from metastasis include persistent knee pain, swelling, and impaired flexion and extension [10]. Patients may experience limited mobility, gait disturbances, and a limp as a result of the cancer burden within the joint. However, in some cases, joint pain may be the sole indication of intra-articular metastases.

In patients with a history of cancer and treatment-resistant unilateral knee pain, metastasis should be considered and promptly identified as a potential cause. As with other bone lesions, skeletal metastases can be challenging to detect on plain radiographs, as more than 50% of bone loss is typically required before changes become radiographically apparent [11]. Therefore, plain films are not always the most effective method for detecting bone metastases, as, unlike primary bone tumors, metastases generally cause little to no periosteal reaction, which is often undetectable on standard radiographs. Other imaging modalities have higher sensitivity in detecting abnormal-to-normal bony changes associated with metastases, with bone scintigraphy achieving (80%), CT (85%), and MRI (90%) [12]. Arthrocentesis of the joint typically yields sanguineous synovial fluid, and cytologic evaluation may reveal malignant cells [13]. Given the rarity of metastatic cancer in the knee, a tissue biopsy is essential for a definitive diagnosis to guide appropriate treatment.

The mechanism underlying joint and synovial metastasis remains uncertain, but two possible theories include hematogenous spread and direct invasion from a metastatic bone lesion. It is important to note that research has demonstrated a strong link between chronic inflammation and the development or progression of malignancies [14]. Chronic inflammation from rheumatoid arthritis could contribute to metastasis by creating a microenvironment that attracts tumor cells, facilitating their homing and growth in inflamed bones. A study using rat models found an increased incidence of breast cancer-associated metastases to the lungs and bones in arthritic mice compared to non-arthritic controls [15]. Elevated levels of pro-inflammatory cytokines, such as interleukin-17, interleukin-6, pro-matrix metallopeptidase 9, insulin-like growth factor-II, and macrophage colony-stimulating factor (M-CSF), were identified in the bone microenvironment of the arthritic mice. These cytokines, along with the inflammatory microenvironment, may play a critical role in facilitating tumor progression and metastasis. The study suggests a potential underlying mechanism contributing to the increased susceptibility of bones to metastases from primary malignancies. Alternatively, angiogenesis, a key process in rheumatoid arthritis, may also contribute to the susceptibility of metastatic malignant cells to establish themselves. Within tumors, angiogenesis has been linked to increased metastatic potential and higher levels of circulating tumor cells [16]. Highly vascularized tissues are more prone to metastasis due to enhanced access to circulating tumor cells and supportive microenvironments [17]. Further research is needed to investigate angiogenesis, inflammatory markers, and tumor-related factors that may influence the development of atypical metastasis sites in individuals with active inflammatory arthritis.

## Conclusions

Metastatic involvement of the knee joint and areas outside the axial skeleton are rare manifestations of lung adenocarcinoma. In patients with known cancer and treatment-refractory isolated monoarthropathy, metastasis should be considered in the differential diagnosis. This case highlights the importance of a comprehensive diagnostic approach in patients with mono-articular joint pain and a history of malignancy to ensure accurate diagnosis and timely management.
